# Randomized, Placebo-controlled Crossover Study of Dalfampridine Extended-release in Transverse Myelitis

**DOI:** 10.1177/2055217317740145

**Published:** 2017-11-08

**Authors:** Kateryna Schwartz, Nicholas F Wymbs, Hwa Huang, Maureen A Mealy, Carlos A Pardo, Kathleen Zackowski, Michael Levy

**Affiliations:** Department of Neurology, 1466 Johns Hopkins University, USA; Department of Neurology, 1466 Johns Hopkins University, USA; 20870 Kennedy Krieger Institute Center for Neurodevelopmental and Imaging Research, USA; Department of Neurology, 1466 Johns Hopkins University, USA; Department of Neurology, 1466 Johns Hopkins University, USA; Department of Neurology, 1466 Johns Hopkins University, USA; Department of Neurology, 1466 Johns Hopkins University, USA; Department of Neurology, 1466 Johns Hopkins University, USA

**Keywords:** 4-aminopyridine, 4AP, transverse myelitis, dalfampridine, Ampyra

## Abstract

**Background:**

Dalfampridine has the potential to be effective in patients with transverse myelitis (TM) as this rare disorder shares some clinical and pathogenic similarities with multiple sclerosis.

**Methods:**

This is a randomized, double-blind, placebo-controlled crossover study of dalfampridine extended-release (D-ER, Ampyra®). Sixteen adult study participants with monophasic TM confirmed by MRI were enrolled if their baseline timed 25-foot walking speed was between 5 and 60 seconds. Participants were randomized to receive 10 mg twice-daily doses of either D-ER or placebo control for eight weeks, then crossed over to the second arm of placebo or dalfampridine for eight weeks. The primary outcome measure was the timed 25-foot walk.

**Results:**

Of 16 enrolled participants, three withdrew and 13 completed the trial. Among the 13 completers, nine individuals showed an average timed walk that was faster in the D-ER arm compared to the placebo arm, but only four participants met the stricter statistical threshold to be classified as a responder. Analyses of secondary clinical outcome measures including strength, balance assessments, spasticity, and Expanded Disability Status Scale (EDSS) score showed trends toward improvement with D-ER.

**Conclusions:**

D-ER may be beneficial in TM to improve walking speed and other neurological functions.

## Introduction

Idiopathic transverse myelitis (TM) is a monophasic autoimmune attack on the spinal cord that leads to weakness, numbness, and bowel/bladder dysfunction. TM differs from multiple sclerosis (MS) as it results from a monophasic-disease pattern, but many patients with TM experience long-lasting neurological symptoms that include weakness and gait disturbance. The incidence is approximately 1.34–4.6 per million per year, with a prevalence of approximately 7500 Americans living with disability from their TM today.^1^ There is a bimodal age distribution with a peak in the teenage years and one in the fourth decade of life, with men and women being equally affected.^2,3^ The etiology of TM is presumed to be an immune-mediated attack two to three weeks following a systemic infection possibly due to molecular mimicry or other presumed immune mechanisms.^4^ TM is conventionally viewed as a sporadic disease, with no strong familial risk factors and no recognized genetic contribution to risk. Treatment is focused on suppressing acute inflammatory process and outcomes are generally favorable with the majority of patients at least recovering the ability to walk with assistance; however, most maintain a long-term impairment in gait function.^5^

Although the pathology of TM has not been adequately characterized as demyelinating, the outcomes of the immune-mediated injury may involve damage of myelinated axons as well as other cord structures. In our clinical experience, many of the gait disturbance and weakness patterns in TM patients resemble those of MS, an observation that suggested using dalfampridine may improve gait in this population. Dalfampridine (also known as fampridine or 4-aminopyridine) is a potassium channel blocker that has been studied since the 1970s for its effects on the nervous system, particularly on amplifying conductivity in demyelinated peripheral nerves fibers, potentiating neurotransmitter release in muscles and increasing post-synaptic action potentials in the spinal cord.^6–9^ The goal of using dalfampridine in demyelinating diseases is to amplify axonal conductance across the demyelinated lesion, which would manifest in improved neurologic function including gait.

In 2010, an extended-release formulation of dalfampridine (D-ER, Ampyra®) was approved by the United States Food and Drug Administration (FDA) to improve walking in patients with MS, as demonstrated by an increase in walking speed. The ER formulation of Ampyra was developed to maintain stable plasma concentrations of the drug and avoid higher peak plasma levels that can lead to seizures.^10^ FDA approval for use in MS is based on a prospectively defined responder cohort analysis, rather than the entire study sample. Between 35% and 42.9% of study participants were classified as responders and on average, this group improved their walking speed by approximately 39%.^11,12^

Our interest in D-ER is focused more narrowly on a subset of patients with TM. In contrast to MS, which affects the multiple central nervous system structures, TM patients have pathological changes restricted to a single lesion in the spinal cord, largely sparing the brain—a factor that may decrease the risk of seizures. In addition to being a potentially safer cohort of patients for D-ER, TM is also a more homogenous disease model in which to test the clinical effects and potentially the mechanism of action of D-ER. This represents the first clinical trial to evaluate the effects of D-ER on TM.

We conducted a clinical trial in idiopathic TM to evaluate the efficacy of D-ER with a primary neurologic outcome of the 25-foot timed walk, a measure of gait impairment, along with several secondary functional and neurophysiological outcome measures. To better understand the mechanisms underlying the proposed functional effects, we used transcranial magnetic stimulation (TMS) as our neurophysiologic measure to identify changes in corticomotor excitability in the spinal cord.

## Methods

**Figure 1.**
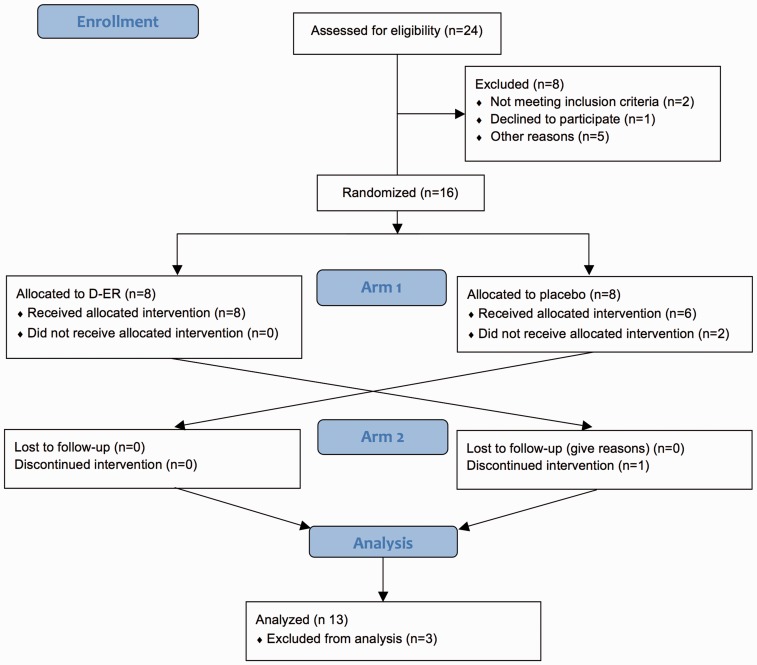
Consort diagram illustrating trial design and recruitment. A total of 24 individuals were screened, of whom 16 were enrolled into the eight-week/arm crossover study. Each arm was preceded by a two-week placebo lead-in and separated by a two-week washout period. Over the course of the study, three people dropped out, with 13 completing the study. CONSORT: Consolidated Standards of Reporting Trials; D-ER: dalfampridine extended-release.

This was a randomized, double-blind, crossover study. Twenty-four adults with monophasic TM, confirmed by magnetic resonance imaging (MRI), with no history of MS or seizure disorder, were screened, of whom 16 were randomized and 13 completed the trial (Figure 1). Inclusion criteria required a history of acute or subacute onset of neurological symptoms, and a change in neurological exam consistent with new or enhancing lesions on MRI. All participants must have remained monophasic for at least three years following onset of disease without treatment, thereby meeting or exceeding the criteria of the 2002 TM Working Group.^13^

After a two-week, single-blinded, placebo lead-in, participants were randomized to receive either 10 mg D-ER or visually identical placebo control tablets for eight weeks. At the conclusion of eight weeks on the initial therapy, individuals completed a two-week wash-out period followed by an additional two-week placebo lead-in and eight weeks of follow-up on the alternate treatment. A final two-week washout period was added after the second treatment phase.

### Efficacy

The primary outcome measure of the trial was the Timed 25-foot walk. The timed 25-foot walk is a quantitative measure of lower extremity function, and was administered by a blinded evaluator. If required, the participant was permitted to use an appropriate assistive device to walk as quickly as he or she could from one end to the other end of a clearly marked, unobstructed, 25-foot course. The task was administered twice, with a standard 1-minute rest period in between, by having the patient walk back the same distance. All participants were tested at the screening visit and at every two weeks during this trial. A “responder” was defined as a participant whose walking speed was faster on at least three of the four timed walks while on treatment than the fastest speed while off treatment. Secondary outcomes assessed the following neurological functions collected at baseline and at the end of each treatment phase:

1. Extremity muscle strength measurements, using a hand-held dynamometer. The specific muscles that were assessed were hip flexors in both the supine and prone positions and grip.
2. Dynamic balance measure: Four-square step test.^14^ This is a standardized test for balance.
3. A 2-minute walk test, which is a standardized test of endurance in MS.^15^
4. The Expanded Disability Status Scale (EDSS), a standardized rating scale assessing overall neurologic function.^16^

### Safety

Baseline hematologic and serologic screening were monitored monthly to rule out undiagnosed kidney, liver, or hematologic diseases that could be exacerbated by the drug and serum human chorionic gonadotropin (hCG) was drawn as necessary to confirm a female participant was not pregnant.

### Neurophysiology

Transcranial magnetic stimulation was assessed once at the start and within 14 days before the end of each treatment period. Surface electromyography was used to measure motor evoked potential response. Participants were seated in a comfortable chair with both arms positioned on a pillow placed on their lap. Electromyography signals were collected using a standard belly-tendon montage with silver chloride electrodes. We monitored upper and lower extremity cortical targets, and recorded from the first dorsal interosseous (FDI) muscle of the hand, and the tibialis anterior muscle (TA) of the leg. Single-pulse stimulation was administered with a Magstim 200^2^ single-pulse stimulator and 70 mm Magstim figure-of-eight coil for FDI, and a 110 mm Magstim double-cone coil for TA. Landmarks for optimal stimulation were captured using a frameless neuronavigation system (Brainsight, www.Rogue-Research.com).

We first determined the resting motor threshold for FDI and TA, following standard procedure, as the minimum stimulator intensity required to evoke a motor evoked potential of at least 50 µV on five out of 10 pulses (Rossini et al., 1994).^17^ Further, an active motor threshold was recorded as the stimulation level to detect evoked amplitudes of at least 200 µV on five of 10 samples following the sustained contraction, measured at 100 µV using continuous electromyographic feedback, of either FDI or TA. By increasing stimulation intensity, we measured corticospinal excitability as the intensity required to produce amplitudes of ∼1 mV (S1mV). We recorded 10 samples for each muscle and activation state (resting, active). Custom MATLAB scripts were used to identify peak-peak amplitude and latency of motor evoked potentials.

### Statistics

For the timed 25-foot walking test, the arithmetic changes of the walking speeds, in feet per second, were analyzed (walking times were converted to speed by taking the reciprocals of the times and multiplying by 25). Analysis of covariance appropriate for a crossover design with baseline as the covariate was performed on the changes from baseline with all the post-treatment visits in a single repeated-measures analysis to robustly account for dropouts after the first treatment arm (assuming the missing data were “missing at random”). An unstructured covariance matrix was allowed with regards to visit but the matrix was constrained to be the same across the treatment arms. The numbers of responders to each treatment were compared via Gart test (Fisher’s exact test comparing the number of individuals responding to only D-ER to those responding to only placebo while controlling for treatment arm). The treatments were compared for the EDSS changes from baseline using repeated-measures proportional odds logistic regression appropriate for a crossover study; an independent working correlation matrix was used. The rest of the secondary clinical outcome measures, including lower extremity muscle strength assessments, Four-square step test, and the 2-minute walk test, were compared in a similar fashion as the Timed 25-foot walk above, except that there was only one post-treatment visit within each of the treatment arms.

## Results

Sixteen individuals enrolled in the study, of whom three withdrew for logistical reasons and 13 completed. Of the 13 completers, six were women, 10 were white, the average age was 55 years (range 33–71), the average duration of disease since onset of TM was 7.2 years and the average EDSS at enrollment was 4.9 (range 3.5–6). The average lesion length was six vertebral segments with a range from two to 13. The demographics and clinical history of the study population are summarized in Table 1.

**Table 1.** Patient demographics.

Demographic	Trial cohort
Number of participants	
Enrolled	16
Withdrew	3
Completed	13
Female sex	46%
Race	
Black	23%
White	77%
Age	
Average (range)	55 (33–71)
Duration of disease	
Average (range)	7.2 (3–31)
EDSS	
Average (range)	4.9 (3.5–6)
MRI lesion locations	
Cervical	4
Cervicothoracic	5
Thoracic	4
MRI lesion length	
Average (range)	6 (2–13)

EDSS: Expanded Disability Status Scale; MRI: magnetic resonance imaging.

**Figure 2.**
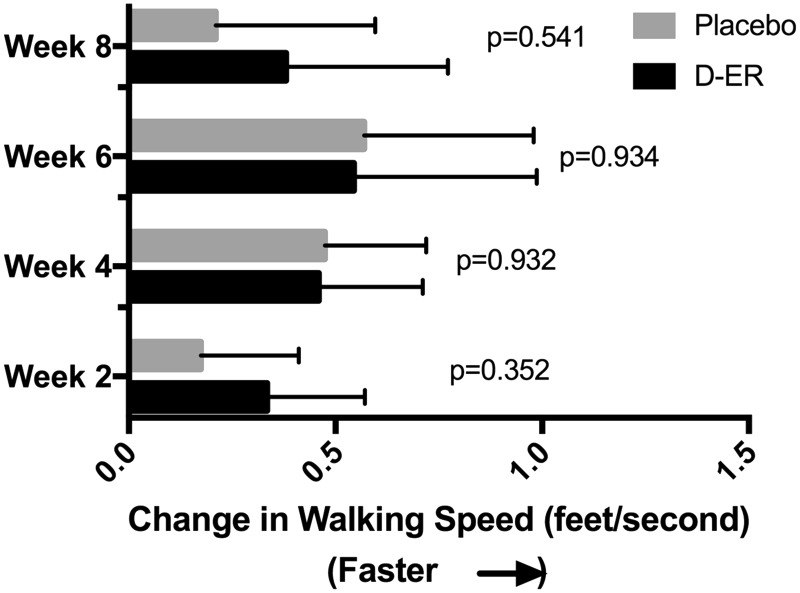
Changes in the primary outcomes, Timed 25-foot walk, over the course of each eight-week period of dalfampridine extended-release (D-ER, black bars) versus placebo (gray bars), measured at two-week intervals. At each time point, both D-ER and placebo led to faster walking speeds with no significant difference between the two periods. The greatest improvements were seen at weeks 4 and 6.

In the primary outcome measure, the Timed 25-foot walk, 11 of the 13 participants showed an average improved walking speed relative to baseline while receiving D-ER and nine of 13 while receiving placebo. At weeks 2 and 8, the improvement was greater in the D-ER arm compared to the placebo arm, but this difference was not significant (Figure 2). At weeks 4 and 6, the improvement relative to baseline was greatest, but again there was no difference compared to placebo. Four participants in the D-ER period met the efficacy threshold for responder. The average improvement over the post-treatment visits among the four D-ER responders was 14.78% faster walking speeds relative to baseline, compared to the only individual who met the efficacy threshold for responder while receiving placebo whose walking speed improved by 0.16% (Gart test for comparing number of responders, *p* = 0.33).

**Figure 3.**
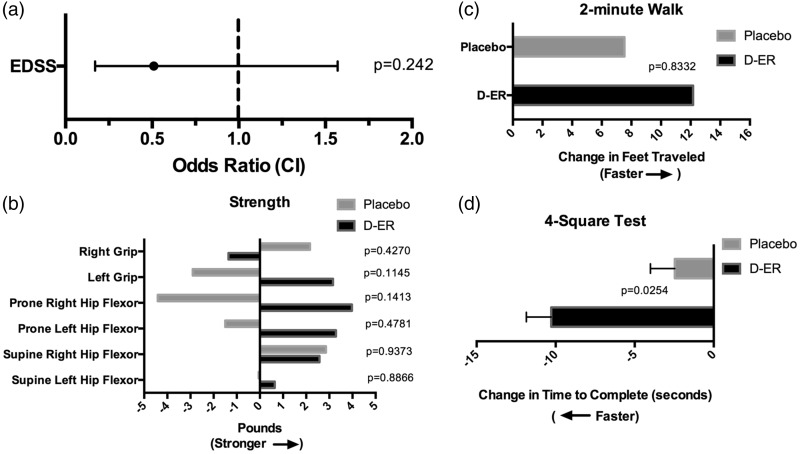
Secondary outcomes. (a) The chance of a higher (i.e. worse) Expanded Disability Status Scale (EDSS) score during the dalfampridine extended-release (D-ER) period was half compared to placebo (*p* = 0.242). (b) Strength in the hips, tested in both prone and supine positions, and grip strength were somewhat stronger in the D-ER period compared to the placebo period, but not statistically significant. (c) Over 2 minutes, participants were able to walk an average of 12 feet farther (± 5 feet) during the D-ER period compared to baseline, whereas participants were able to walk an average of 7.5 feet farther (± 3 feet) during the placebo period (not statistically significant). (d) In the Four-square test for balance, participants were able to complete the test 10 seconds faster during the D-ER period compared to 2 seconds faster in the placebo period (error bars represent SEM, *p* = 0.0254). CI: confidence interval.

Analysis of secondary outcome measures compared each subject’s EDSS, strength, 2-minute walk and 4-square balance test in the D-ER period with the placebo period. For the EDSS, the ratio of the odds of a higher versus a lower change from baseline score with D-ER relative to placebo was 0.51 (Figure 3(a), *p* = 0.242). Strength in the hips and hands were somewhat stronger in the D-ER period compared to placebo (Figure 3(b)), and while participants were in the D-ER period, on average they walked 5 feet farther in 2 minutes than while in the placebo period (Figure 3(c)). On balance assessment, participants demonstrated improved balance on the 4-square test while on D-ER compared to placebo (Figure 3(d), *p* = 0.025). While all of these secondary measures trended toward improvement in the D-ER period, none were statistically significant except the 4-square test for balance.

There were no serious adverse events in the trial. Compared to placebo, individuals taking D-ER reported more insomnia and complaints of weakness (Table 2), and there were no metabolic abnormalities in either group during the course of the study. Increased spasticity and neuropathic pain due to D-ER were not observed or reported.

**Table 2.** Adverse events reported during the two eight-week treatment periods of the crossover, by percent of patients reporting.

Adverse events	D-ER	Placebo
Urinary urgency	23%	31%
Insomnia	15%	8%
Dizziness/Imbalance	0%	15%
Subjective weakness	31%	15%
Fatigue	31%	31%
Muscle stiffness	31%	38%

D-ER: dalfampridine extended-release.

At baseline, TMS revealed a prolonged latency without changes in the amplitude across the TM lesion, consistent with a demyelinating phenotype. In response to D-ER, there were no observable changes to latency or amplitude in either the resting or activated state of the TA or FDI muscles, even when results were isolated to the responder subgroup (Table 3).

**Table 3.** Transcranial magnetic stimulation (TMS) amplitudes and latencies.

TMS	Baseline	D-ER	Placebo
FDI			
Resting			
Amplitude (mean, mV)	0.80	0.99	0.87
Latency (mean, ms)	24.86	24.85	24.68
Activated			
Amplitude (mean, mV)	1.20	0.99	0.87
Latency (mean, ms)	24.04	23.61	24.69
TA			
Resting			
Amplitude (mean, mV)	0.21	0.26	0.34
Latency (mean, ms)	38.68	38.43	40.51
Activated			
Amplitude (mean, mV)	0.49	0.45	0.55
Latency (mean, ms)	41.10	39.94	40.71

FDI: first dorsal interosseous; TA: tibialis anterior muscle; D-ER: dalfampridine extended-release.

## Discussion

We conducted a randomized, double-blind, placebo-controlled crossover study of D-ER in a cohort of participants with TM. The rationale for this trial was based on experiential evidence that D-ER may improve gait function in a subset of patients with TM. The rationale was further bolstered by our initial baseline TMS interrogation of the motor tracts, which suggested the pathology involved myelinated pathways and may therefore respond favorably to D-ER therapy (Wymbs et al., in preparation). Despite an average duration of disease of 10 years since the TM attack initially occurred, we found that the drug showed a trend in improving walking speeds in 85% of individuals while receiving D-ER versus 69% while receiving placebo, and met the higher threshold for responder in 33% while receiving D-ER versus only 8% while receiving placebo, although, given the small numbers of participants, this percentage did not reach statistical significance (*p* = 0.33).

The efficacy trends in this trial of TM participants are similar to two early small trials in MS. The first randomized, double-blind, placebo-controlled trial of D-ER with MS patients in 1997 enrolled 10 patients and found that nine of them had improvements in walking speeds by an average of 22%.^18^ The second randomized, placebo-controlled study in MS did not meet its pre-defined endpoint, but a post-hoc responder analysis identified 38% of patients on D-ER (and 8% on placebo) who met the threshold for responder.^18^ These results in MS are similar to our findings in the TM patient population and likely suggest that larger studies might have yielded similar positive outcomes with increased statistical power. D-ER has been tested for benefits in other neurological functions besides walking, including cognition,^19^ fatigue,^20^ mood,^21^ visual acuity,^22^ nystagmus, dexterity and strength in the arms,^23^ balance,^24^ and language.^25^ The trends toward improvement observed in these trials are similar to trends seen for some of the secondary outcomes in this study, including endurance, strength and balance.

This study was limited in the ability to recruit a homogenous patient population as TM is a rare disease with a heterogeneous presentation depending on the degree and location of spinal cord damage. Each visit involved several physically demanding tests that are fatiguing to different degrees for each patient. Some of these limitations were accounted for in the crossover design of the trial, but because of the length of the study, recruitment was somewhat difficult and a number of screen failures and withdrawals occurred. Future trials of D-ER in TM might be expanded to include related TM disorders such as neuromyelitis optica spectrum disorders.

## Conclusions

D-ER showed trends for improvement in patients with TM in a number of measures, including gait speed and balance, with no new safety signals. A larger study in this patient population with TM would be required to further examine such effects and determine their significance.

## References

[1] Kaplin AI Krishnan C Deshpande DM et al. Diagnosis and management of acute myelopathies. Neurologist 2005; 11: 2–18. 15631640 10.1097/01.nrl.0000149975.39201.0b

[2] Bhat A Naguwa S Cheema G et al. The epidemiology of transverse myelitis. Autoimmun Rev 2010; 9: A395–A399. 20035902 10.1016/j.autrev.2009.12.007

[3] Krishnan C Kaplin AI Deshpande DM et al. Transverse myelitis: Pathogenesis, diagnosis and treatment. Front Biosci 2004; 9: 1483–1499. 14977560 10.2741/1351

[4] Frohman EM Wingerchuk DM Clinical practice. Transverse myelitis. N Engl J Med 2010; 363: 564–572. 20818891 10.1056/NEJMcp1001112

[5] Greenberg BM Thomas KP Krishnan C et al. Idiopathic transverse myelitis: Corticosteroids, plasma exchange, or cyclophosphamide. Neurology 2007; 68: 1614–1617. 17485649 10.1212/01.wnl.0000260970.63493.c8

[6] Jankowska E Lundberg A Rudomin P et al. Effects of 4-aminopyridine on transmission in excitatory and inhibitory synapses in the spinal cord. Brain Res 1977; 136: 387–392. 200308 10.1016/0006-8993(77)90816-2

[7] Lundh H Thesleff S The mode of action of 4-aminopyridine and guanidine on transmitter release from motor nerve terminals. Eur J Pharmacol 1977; 42: 411–412. 15849 10.1016/0014-2999(77)90176-5

[8] Molgo J Lemeignan M Lechat P Changes in transmitter release at frog neuromuscular junction induced by 4-aminopyridine [article in French]. C R Acad Sci Hebd Seances Acad Sci D 1975; 281: 1637–1639. 3292

[9] Sherratt RM Bostock H Sears TA Effects of 4-aminopyridine on normal and demyelinated mammalian nerve fibres. Nature 1980; 283: 570–572. 7354839 10.1038/283570a0

[10] Hayes KC Katz MA Devane JG et al. Pharmacokinetics of an immediate-release oral formulation of Fampridine (4-aminopyridine) in normal subjects and patients with spinal cord injury. J Clin Pharmacol 2003; 43: 379–385. 12723458 10.1177/0091270003251388

[11] Goodman AD Brown TR Edwards KR et al. A phase 3 trial of extended release oral dalfampridine in multiple sclerosis. Ann Neurol 2010; 68: 494–502. 20976768 10.1002/ana.22240

[12] Goodman AD Brown TR Krupp LB et al. Sustained-release oral fampridine in multiple sclerosis: A randomised, double-blind, controlled trial. Lancet 2009; 373: 732–738. 19249634 10.1016/S0140-6736(09)60442-6

[13] Myelitis Consortium Working Group Transverse Proposed diagnostic criteria and nosology of acute transverse myelitis. Neurology 2002; 59: 499–505. 12236201 10.1212/wnl.59.4.499

[14] Blennerhassett JM Jayalath VM The Four Square Step Test is a feasible and valid clinical test of dynamic standing balance for use in ambulant people poststroke. Arch Phys Med Rehabil 2008; 89: 2156–2161. 18996245 10.1016/j.apmr.2008.05.012

[15] Gijbels D Eijnde BO Feys P Comparison of the 2- and 6-minute walk test in multiple sclerosis. Mult Scler 2011; 17: 1269–1272. 21642370 10.1177/1352458511408475

[16] Kurtzke JF Rating neurologic impairment in multiple sclerosis: An Expanded Disability Status Scale (EDSS). Neurology 1983; 33: 1444–1452. 6685237 10.1212/wnl.33.11.1444

[17] Rossini PM, Barker AT, Berardelli A, et al. Non-invasive electrical and magnetic stimulation of the brain, spinal cord and roots: basic principles and procedures for routine clinical application. Report of an IFCN committee. *Electroencephalogr Clin Neurophysiol* 1994; 91: 79–92. 10.1016/0013-4694(94)90029-9 7519144

[18] Schwid SR Petrie MD McDermott MP et al. Quantitative assessment of sustained-release 4-aminopyridine for symptomatic treatment of multiple sclerosis. Neurology 1997; 48: 817–821. 9109861 10.1212/wnl.48.4.817

[19] Triche EW Ruiz JA Olson KM et al. Changes in cognitive processing speed, mood, and fatigue in an observational study of persons with multiple sclerosis treated with dalfampridine-ER. Clin Neuropharmacol 2016; 39: 73–80. 26818040 10.1097/WNF.0000000000000130

[20] Morrow SA Rosehart H Johnson AM The effect of Fampridine-SR on cognitive fatigue in a randomized double-blind crossover trial in patients with MS. Mult Scler Relat Disord 2017; 11: 4–9. 28104253 10.1016/j.msard.2016.10.011

[21] Pavsic K Pelicon K Ledinek AH et al. Short-term impact of fampridine on motor and cognitive functions, mood and quality of life among multiple sclerosis patients. Clin Neurol Neurosurg 2015; 139: 35–40. 26363365 10.1016/j.clineuro.2015.08.023

[22] Horton L Conger A Conger D et al. Effect of 4-aminopyridine on vision in multiple sclerosis patients with optic neuropathy. Neurology 2013; 80: 1862–1866. 23616154 10.1212/WNL.0b013e3182929fd5 PMC3908347

[23] Claassen J Spiegel R Kalla R et al. A randomised double-blind, cross-over trial of 4-aminopyridine for downbeat nystagmus—effects on slowphase eye velocity, postural stability, locomotion and symptoms. J Neurol Neurosurg Psychiatry 2013; 84: 1392–1399. 23813743 10.1136/jnnp-2012-304736

[24] Hupperts R Lycke J Short C et al. Prolonged-release fampridine and walking and balance in MS: Randomised controlled MOBILE trial. Mult Scler 2016; 22: 212–221. 25921050 10.1177/1352458515581436 PMC4749757

[25] Magnin E Sagawa Y Jr Chamard L et al. Verbal fluencies and fampridine treatment in multiple sclerosis. Eur Neurol 2015; 74: 243–250. 26624899 10.1159/000442348

